# Characteristics of patients receiving midwife-led prenatal care in Canada: results from the Maternity Experiences Survey (MES)

**DOI:** 10.1186/s12884-017-1350-4

**Published:** 2017-06-02

**Authors:** Peri Abdullah, Sabrina Gallant, Naseem Saghi, Alison Macpherson, Hala Tamim

**Affiliations:** Kinesiology and Health Science, York University, 4700 Keele Street, Toronto, ON M3J 1P3 Canada

**Keywords:** Midwifery, Canada, Education, Alcohol, Pregnancy

## Abstract

**Background:**

The aim of this study was to determine the characteristics of women in Canada who received care from a midwife during their prenatal period.

**Methods:**

The findings of this study were drawn from the Maternity Experiences Survey (MES), which was a cross-sectional survey that assessed the experiences of women who gave birth between November 2005 and May 2006. The main outcome variable for this study was the prenatal care provider (i.e. midwife versus other healthcare providers). Demographic, socioeconomic, as well as health and pregnancy factors were evaluated using bivariate and multivariate models of logistic regression.

**Results:**

A total of 6421 participants were included in this analysis representing a weighted total of 76,508 women. The prevalence of midwife-led prenatal care was 6.1%. The highest prevalence of midwife-led prenatal care was in British Columbia (9.8%), while the lowest prevalence of midwife-led prenatal care was 0.3% representing the cumulative prevalence in Nova Scotia, Prince Edward Island, Newfoundland and Labrador, New Brunswick, Saskatchewan, and Yukon. Factors showing significant association with midwife-led prenatal care were: Aboriginal status (OR = 2.26, 95% CI: 1.41–3.64), higher education with bachelor and graduate degree attainment having higher ORs when compared to high-school or less (OR = 2.71, 95% CI: 1.71–4.31 and OR = 3.17, 95% CI: 1.81–5.55, respectively), and alcohol use (OR = 1.63, 95% CI: 1.17–2.26). Age, marital status, immigrant status, work during pregnancy, household income, previous pregnancies, perceived health, maternal Body Mass Index (BMI), and smoking during the last 3 months of pregnancy were not significantly associated with midwife care.

**Conclusions:**

In general, women who were more educated, have aboriginal status, and/or are alcohol drinkers were more likely to receive care from midwives. Since MES is the most recent resource that includes information about national midwifery utilization, future studies can provide more up-to-date information about this important area. Moreover, future research can aim at understanding the reasons that lead women to opt for midwife-led prenatal care.

## Background

According to the International Confederation of Midwives, the World Health Organization, and the International Federation of Gynecology and Obstetrics: “the midwife is recognized as a responsible and accountable professional who works in partnership with women to give the necessary support, care and advice during pregnancy, labour and the post-partum period, to conduct births on the midwife’s own responsibility and to provide care for the newborn and the infant” [1]. Midwives are available in at least 108 countries around the world, spanning the Americas, Europe, Africa and Asia Pacific [2]. While different combinations of midwife-led continuity, medical-led and shared models of care exist in some developed countries (e.g. Australia, New Zealand, the Netherlands, the United Kingdom and Ireland), medical doctors remain the primary care providers for the majority of childbearing patients in North America [3].

A Cochrane review performed in 2015 showed that patients of midwife-led continuity model of care (where the midwife is the primary care provider from initial booking until the end of the post-natal period) were less likely to experience the following as compared to other models of care (including obstetrician provided care, family doctor provided care, and shared care): epidural/spinal analgesia, forceps/vacuum vaginal birth, preterm births, amniotomy, episiotomy, fetal loss and neonatal death [3]. Patients in the midwife-led continuity model were also more likely to experience spontaneous vaginal birth and longer duration of labour as compared to the other models of care [3]. No significant differences were found between the different models of care in caesarean birth, antepartum and postpartum hemorrhage, antenatal hospitalization, labour induction, breastfeeding initiation, mean hospital stay and low birth weight [3]. Therefore, the authors concluded that midwife led care shows no harmful effects and presents some important benefits [3]. Moreover, the midwife-led continuity model shows a trend towards cost-effectiveness when compared to the medical-led care [3].

In Canada, midwives are considered autonomous primary health care professionals providing prenatal care, care during labour and birth and postnatal care [4]. Depending on regional availability, midwives are able to deliver their care in any birth setting including hospitals, birth centres, health clinics or the patient’s home [4]. There are currently over 1500 practicing midwives throughout most regions in Canada, the majority of whom are practicing in Ontario. However, according to the Maternity Experiences Survey (MES), only 6.1% of women in Canada received midwife-led prenatal care in 2005/2006 [5]. More recently, the Canadian Association of midwives has reported that in 2015/2016 only 9.8% of Canadian births were performed with the assistance of a midwife [6]. This underutilization of midwifery in Canada has been attributed to various reasons including: low birth rate, national health insurance for physician care and the high number of physicians and nurses practicing in Canada [7].

Current knowledge of the characteristics of women receiving midwife-led prenatal care and birth is very limited, but generally shows regional variability. In the United States, studies have shown that midwives mostly serve less educated, non-White, teenage mothers [8, 9]. On the other hand, a study in the Netherlands, showed that women who are of other European origin or of Asian origin were more likely than Dutch women to initiate care with a midwife [10]. In addition, a study about birthing services in Victoria, Australia showed that women living in a rural area were slightly more likely to attend midwife clinics than metropolitan hospitals (5.3 and 2.8%, respectively) [11].

In Canada, two studies have reported characteristics of women receiving midwife-led prenatal care [12, 13]. A study performed in Manitoba assessed the trends of midwifery use in the province and found that multiparous women who are between the ages of 20–34 years were more likely to receive midwife-led prenatal care [12]. The second study was performed by Klein et al. on a more national scale with an objective to investigate the attitudes on nulliparous women to birth technology [13]. However, the sample in that study was not representative of the Canadian population due to the sampling method used [13]. Nevertheless, the authors reported that women receiving midwife-led prenatal care were slightly older [13]. Therefore, no representative national-scale studies were found, which describe the characteristics of women receiving midwife-led prenatal in Canada. The main purpose of this study was to evaluate the characteristics of these patients compared with those who receive their care from other models on a Canada-wide scale.

## Methods

The present study is a secondary data analysis of the Maternity Experiences Survey (MES), which was performed by Statistics Canada between November 2005 and May 2006. This survey was conducted following the Canadian Census of population, and the sample was selected using information collected in the census. Access to MES data was granted by the Research Data Center (RDC) at York University. The survey was cross-sectional and targeted women who gave birth between February 15 and May 15, 2006 in all Canadian provinces, or between November 1, 2005 and February 1, 2006 in the territories [14]. Moreover, the women had to have had a single birth, were at least 15 years of age at the time of birth and whose babies were born in Canada and lived with the mother no less than one night per month [14]. Women who lived in collective dwellings or on First Nations reserves were excluded [14]. The final sample included 8542 respondents, 6421 of which granted Statistics Canada permission to share their responses [14]. Data collection was done through a Computer Assisted Telephone Interview on the provincial level [14]. If a telephone interview was not possible in the territories, personal interviews were performed with a paper copy of the questionnaire [14]. The average time for the interview was 45 min [14]. Responding to the survey was voluntary, with a response rate of 78% [14]. Details about the design and methodology of MES have been described previously [15]. The protocol of MES has been reviewed by the Health Canada’s Science Advisory Board and Research Ethics Board and the Federal Privacy Commissioner, and approved by the Statistics Canada’s Policy Committee. Since this project was based on secondary data analysis of MES, institutional ethics approval was not required.

For the present study, the main outcome was defined as midwife-led prenatal care asked by the question “From which type of healthcare provider, such as an obstetrician, family doctor or midwife, did you receive most of [your prenatal] care?”. Possible responses to this question were: Obstetrician, Gynecologist, OBGYN, family doctor, General Practitioner (GP), doctor, midwife, nurse or nurse practitioner or other. For the purposes of this study, the outcome variable was dichotomized and the two levels were ‘midwife-led prenatal care’ and ‘other’.

The following variables were evaluated at each level of the outcome variable: Maternal age (three levels: <20, 20–34 and 35+ years of age), aboriginal status, mother’s highest level of education (four levels: high-school or less, post-secondary below bachelor, bachelor degree and graduate degree), mother’s employment status during pregnancy, total annual household income (four levels: <30,000, 30,000–<60,000, 60,000–<100,000 and 100,000 or more Canadian Dollars), marital status (two levels: with partner, without partner), immigration status (two levels: has/had immigrant status and never had immigrant status), self-reported alcohol use during pregnancy, smoking during the last 3 months of pregnancy, perceived health of the mother (three levels: excellent/very good, good and fair/poor), Body Mass Index (BMI) recoded according to Health Canada guidelines [16] (four levels: underweight, normal weight, overweight and obese) and whether this was the mother’s first pregnancy.

Bivariate logistic regressions were used to assess the relationship between the outcome ‘midwife-led prenatal care’ and the covariates. Odds Ratios (ORs) and 95% Confidence Intervals (95% CIs) were obtained to assess the relationship between each of the covariates and the outcome variable. A multivariate logistic regression model was also created with the same outcome ‘midwife-led prenatal care’ and the independent variables being all the covariates mentioned above. This resulted in adjusted ORs and 95% CIs for the outcome variable at each covariate. Survey weights were applied to each variable and calculated estimate, making the data more representative of the Canadian population as a whole. Bootstrapping was performed to account for the complex design of the survey. All the analyses were computed in the Stata Statistical Software version 13 (StataCorp, College Station, TX).

## Results

**Fig. 1:**
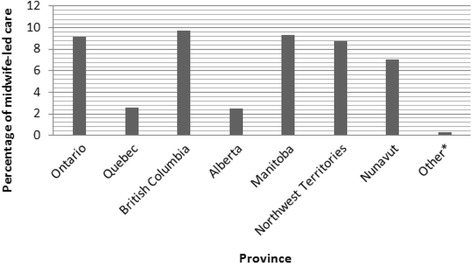
Frequency of midwife-led prenatal care across different provinces In Canada. * ‘Other’ includes: Nova Scotia, Prince Edward Island, Newfoundland and Labrador, New Brunswick, Saskatchewan and Yukon. These provinces were grouped together due to low cell counts. Data source: Maternity Experiences Survey (MES)

The total number of participants included in this analysis was 6421 participants representing a weighted total of 76,508 women. By province, women residing in British Columbia were more likely to receive midwife-led care at 9.8%, followed by Manitoba (9.4%) and Ontario (9.2%) (Fig. 1). The lowest prevalence of midwife-led care was 0.3% which represented the total prevalence in all of: Nova Scotia, Prince Edward Island, Newfoundland and Labrador, New Brunswick, Saskatchewan, and Yukon (Fig. 1).

The results of the bivariate and multivariate analyses are shown in Table 1. Multivariate logistic regression analysis showed that the significant factors associated with receiving care from a midwife were: Aboriginal status (OR = 2.26, 95% CI: 1.41–3.65), bachelor and graduate degree attainment when compared to high-school or less (OR = 2.71, 95% CI: 1.71–4.31 and OR = 3.17, 95% CI: 1.81–5.55, for bachelor and graduate level, respectively), and self-reported alcohol use (OR = 1.63, 95% CI: 1.17–2.26) (Table 1). Factors that were not significantly associated with midwife-led care were age, marital status, perceived health, maternal BMI before pregnancy, whether this was the first pregnancy for that mother, ever having had an immigrant status, and working during pregnancy (Table 1).

**Table 1:** Prevalence, ORs and 95% CIs of women receiving midwife-led care in Canada

Predictor	Total sample size (weighted)	Mid-wife led prenatal care			
		Sample size (weighted)	%^a^	Unadjusted OR (95% CI)	Adjusted OR (95% CI)
Total	76,085	4609	6.1		
Demographic variables					
Maternal age at birth (years)					
< 20	1504	68	4.6	1.00	1.00
20–34	59,115	3154	5.4	1.19 (0.56–2.49)	0.76 (0.31–1.89)
35 and above	15,890	1388	8.8	2.01 (0.93–4.35)	1.19 (0.46–3.01)
Aboriginal Status					
Yes	3224	315	9.8	1.75 (1.14–2.67)	2.26 (1.41–3.65)
No	72,910	4256	5.9	1.00	1.00
Marital Status					
With partner	69,833	4294	6.2	1.37 (0.88–2.14)	1.13 (0.65–1.96)
No partner	6374	291	4.6	1.00	1.00
Ever had immigrant status					
Yes	16,764	864	5.2	0.82 (0.61–1.09)	0.72 (0.51–1.01)
No	59,337	3709	6.3	1.00	1.00
Socio-economic variables					
Maternal highest level of education					
Highschool or less	15,836	555	3.5	1.00	1.00
Post-secondary, below Bachelor	32,914	1650	5.0	1.45 (1.01–2.08)	1.52 (0.99–2.35)
Bachelor	19,429	1632	8.5	2.52 (1.76–3.60)	2.71 (1.71–4.31)
Graduate degree	7444	750	10.1	3.06 (1.96–4.76)	3.17 (1.81–5.55)
Maternal work status during pregnancy					
Yes	52,612	3089	5.9	0.92 (0.73–1.16)	0.76 (0.58–1.00)
No	23,555	1497	6.4	1.00	1.00
Total household income					
<$ 30,000	12,288	696	5.7	1.00	1.00
$ 30,000–<$ 60,000	22,080	1223	5.6	0.97 (0.69–1.38)	0.82 (0.54–1.23)
$60,000–<$ 100,000	23,116	1323	5.8	1.01 (0.71–1.43)	0.66 (0.43–1.02)
$ 100,000 and above	14,446	1147	8.0	1.43 (0.99–2.06)	0.72 (0.45–1.16)
Health and pregnancy variables					
First pregnancy					
Yes	25,071	1520	6.1	1.01 (0.80–1.28)	1.21 (0.93–1.56)
No	51,149	3082	6.0	1.00	1.00
Perceived health					
Excellent/very good	55,395	3636	6.6	1.56 (0.87–2.79)	1.79 (0.87–3.69)
Good	16,988	798	4.7	1.09 (0.58–2.05)	1.41 (0.66–3.00)
Fair/poor	4070	176	4.3	1.00	1.00
Maternal BMI before pregnancy					
Underweight (<18.5)	4588	190	4.1	0.62 (0.34–1.12)	0.73 (0.38–1.41)
Normal weight (18.5–24.99)	44,584	2890	6.5	1.00	1.00
Overweight (25–29.99)	15,788	1019	6.5	0.99 (0.75–1.32)	1.06 (0.79–1.43)
Obese (30 and above)	10,186	417	4.1	0.61 (0.41–0.91)	0.65 (0.42–1.01)
Smoking during the last 3 months of pregnancy					
Yes	8016	366	4.6	0.73 (0.49–1.09)	0.86 (0.52–1.42)
No	68,320	4236	6.2	1.00	1.00
Alcohol use during pregnancy					
Yes	7964	768	9.7	1.78 (1.30–2.44)	1.63 (1.17–2.26)
No	68,081	3834	5.7	1.00	1.00

^a^Row percentage of midwife-led prenatal care

Data Source: Maternity Experiences Survey (*MES*). *OR* Odds Ratio, *CI* Confidence Interval

## Discussion

In this study, we investigated the characteristics of women receiving prenatal care from midwives on a national scale in Canada. Our results show that receiving midwife-led care during pregnancy was influenced most notably by higher levels of education, aboriginal status, and self-reported alcohol use during pregnancy. Moreover, the prevalence of midwife-led prenatal care varies greatly by province, being highest in British Columbia at 9.8% and lowest in all of: Nova Scotia, Prince Edward Island, Newfoundland and Labrador, New Brunswick, Saskatchewan, and Yukon (combined prevalence: 0.32%). This study is one of the first to describe the fundamental characteristics of women in Canada who receive prenatal care from a midwife and highlights the importance of gaining more up-to-date statistics of midwifery utilization.

Based on the findings of the present study, the three provinces with the highest uptake of midwife-led prenatal care were British Columbia (9.8%), followed by Manitoba (9.4%) and Ontario (9.2%). However, in general there has been an increase in the uptake of midwife-led prenatal care; nationally increasing from 6.1% in 2005/2006 [5] to 9.8% in 2015/2016 [6]. As of 2016 the three provinces with the highest uptake of midwife-led prenatal care was as follows: British Columbia (21%), Nunavut (15.4%) and Ontario (15.2%), while Manitoba’s uptake has now dropped to 6.5% [6]. Midwifery in Manitoba has been regulated and publicly funded since the year 2000 [17], however, there have been some setbacks in recent years where the newly elected provincial government opted to cancel the existing joint educational program [6]. This could have contributed to the decline in midwife-led prenatal care in Manitoba from 2005/2006 to 2015/2016. On the other hand, the rise in midwife-led prenatal care in Nunavut during that period can be explained by the later regulation of midwifery as a profession in that region, having only been regulated since 2011 [6]. In addition to Nunavut, the following provinces have become regulated since the MES data collection, potentially contributing to the observed increase in the national uptake of midwife-led prenatal care: Saskatchewan, Nova Scotia, Newfoundland and Labrador, and New Brunswick [6]. Midwifery in Prince Edward Island and Yukon Territory currently lacks a regulating body [6]. Moreover, it seems that with regulation comes funding, and midwifery is now covered by provincial/territorial health care plans in most regions of Canada, potentially increasing accessibility to this service [18]. On the other hand, midwifery is not covered by the health care plan in Prince Edward Island [19]. Unfortunately, no information about midwifery coverage was found for New Brunswick, Newfoundland and Labrador, and Yukon territory.

In terms of demographic characteristics, having aboriginal status was associated with increased likelihood of choosing a midwife. Aboriginal individuals are less likely to seek medical attention through government-run health care systems in Canada, due to factors such as social inequality, decreased specialist referrals, lowered access to higher-tier medical therapies, and decreased standards of medical care (both perceived and actual) [20]. Aboriginal communities are also more likely to look for alternative therapies, perhaps as a result of disintegration throughout the non-aboriginal Canadian community, or as a complement to the increased importance placed on spiritual practices; however, data on the use of this population is under-represented [21].

In terms of socioeconomic factors, higher levels of education, was associated with increased use of midwives. Looking solely at education, previous literature has shown that university-educated women are more likely to feel in charge of their own health, perhaps due to increased education leading to a more keen interest in their own health status [22, 23]. Midwifery programs focusing solely on a more highly-educated demographic may choose to incorporate health literacy as a core component, which may serve to keep their patient demographic involved in their own care and emotionally engaged about the process throughout [24].

In terms of health factors, this study found that alcohol use during pregnancy is associated with higher odds of receiving prenatal care from a midwife. For the purposes of this study, this variable was dichotomoized which may mask the effects of heavy drinking versus light drinking during pregnancy. If this association does exist, however, it may be because alcohol users prefer to avoid using physicians and the national healthcare system for fear of being judged by the physicians, nurses and staff. Alternatively, women receiving care from a midwife may feel more comfortable disclosing their habits (alcohol or otherwise) to one individual. Moreover, alcohol users may not be able to uphold a doctor-patient relationship and may prefer the flexibility of a midwife. Future studies can further investigate the association between alcohol use during pregnancy and receiving prenatal care from a midwife.

The main limitation of this study is that the data used is 10 years old. However, up to the authors’ knowledge, the MES is the most recent database that provides information about the characteristics of midwife-led prenatal care on a national scale in Canada. Another limitation is the cross-sectional design of the survey, which may introduce reverse causality. In addition, with self-reported data, the possibility of information bias should be considered, especially for alcohol use and smoking during pregnancy where respondents may not feel encouraged to provide accurate answers that may present themselves unfavourably. Other contributors to information bias include lack of recall and closed-ended questions, which have a lower validity rate than other question types and the answer options could lead to unclear data due to differences in interpretation by different respondents (for example, the answer option “somewhat agree” may elicit different responses). Last but not least, this study did not control for all of the confounding variables that could have contributed to the associations we found. These include midwife-led care in previous pregnancies and ethnicity. Regardless of these limitations, this study used a large dataset which studied a large amount of predictors and was weighted to represent the Canadian population of pregnant women. This survey also had a high response rate of 78%. As far as the authors are aware, this is the first study that aims to describe the characteristics of women choosing midwife-led prenatal care in Canada on a national scale.

## Conclusions

In conclusion, receiving prenatal care from a midwife in Canada is associated with increased education, having an aboriginal status, and consuming alcohol during pregnancy. The prevalence of midwife-led prenatal care also varies greatly by province. Up to the authors’ knowledge, this is the first study that investigates the characteristics of women receiving midwife-led prenatal care in Canada. Further research can provide in-depth understanding of factors that play a role in the increased utilization of midwives during pregnancy and can provide useful clinical practice guidelines to ensure high-quality patient-oriented care, tailored to individual patient preferences and specific circumstances. In addition, future studies and national efforts can provide more up to date information about the characteristics of women receiving midwife-led prenatal care in Canada, especially since midwife-led prenatal care seems to be on the rise in Canada with fluctuating trends in some provinces.

## Abbreviations

95% CI: 95% Confidence Interval
BMI: Body Mass Index
MES: Maternity Experiences Survey
OR: Odds Ratio
RDC: Research Data Centre
